# Severe Gastrointestinal Bleeding Due to Painless Intermittent Ileal Intussusception—A Case Report

**DOI:** 10.1002/ccr3.9571

**Published:** 2024-11-15

**Authors:** Leonard Fehring, Sarah Krüger, Sarah Sass, Sophia Vorreuther, Sophie Vieser, Hendrik Brinkmann, Lars Kamper, Maximilian Ackermann, Christian Prinz

**Affiliations:** ^1^ Fakultät für Gesundheit Universität Witten/Herdecke Witten Germany; ^2^ Klinik für Gastroenterologie HELIOS Universitätsklinikum Wuppertal Wuppertal Germany; ^3^ Zentrum für Radiologie HELIOS Universitätsklinikum Wuppertal Wuppertal Germany; ^4^ Institut für Pathologie HELIOS Universitätsklinikum Wuppertal Wuppertal Germany

**Keywords:** capsule endoscopy, gastrointestinal hemorrhage, leiomyomas, small intestine

## Abstract

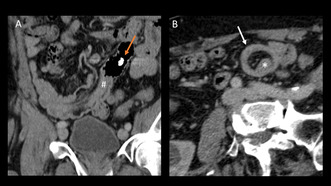

**Summary**

- Ileal intussusceptions caused by leiomyomas can present as intermittent, painless gastrointestinal bleedings.
- Ensuring the proper passage of a video capsule is crucial before initiating capsule endoscopy.
- To distinguish between leiomyoma and leiomyosarcoma the proliferation rate is important and has an impact on the further treatment, classification and disease progression.

## 1 Introduction

Acute gastrointestinal hemorrhage poses a significant challenge for healthcare providers, as bleeding can originate from various parts of the digestive tract and vary in severity, from subtle to life‐threatening. Thus, a prompt assessment and efficient, targeted diagnostic is critical. While most bleeding sources can be identified and treated endoscopically further diagnostic procedures and treatments are occasionally necessary. Here, we present a case of severe gastrointestinal bleeding caused by intermittent ileal intussusception due to a leiomyoma. We depict and explain the diagnostic findings typical of intussusception and highlight potential pitfalls. Additionally, we explain how to distinguish between leiomyomas and leiomyosarcomas histologically and why it is important to do so.

Intussusceptions are defined as the invagination of one segment of the bowel into an adjacent segment of the bowel [1]. In adults, intussusception is rare, accounting for only 1%–5% of bowel obstructions [2, 3] and occurring in only 0.08% of all abdominal surgeries [4]. Surgical resection of the causative lesion is the recommended treatment [5]. The median age of presentation is 50 years, with no gender preference [1]. Most cases occur in the small intestine, typically caused by altered bowel mechanics due to a malignant or benign tumor serving as a lead point [1].

Benign tumors of the small intestine are very rare, comprising 1%–6% of all gastrointestinal neoplasms. Leiomyomas account for 20% of these benign tumors, typically occur in individuals between the ages of 50 and 60 [6] and originate from smooth muscle tissue.

Macroscopically, leiomyomas can develop into painful, hard nodules. Common localizations include the subcutaneous tissue, smooth muscle layers of internal organs, and blood vessel walls. The most common site in the intestine is the jejunum, followed by the ileum and duodenum [7]. Four different growth patterns have been described: intraluminal, intramural, extraluminal, and dumbbell‐shaped [8].

**FIGURE 1:**
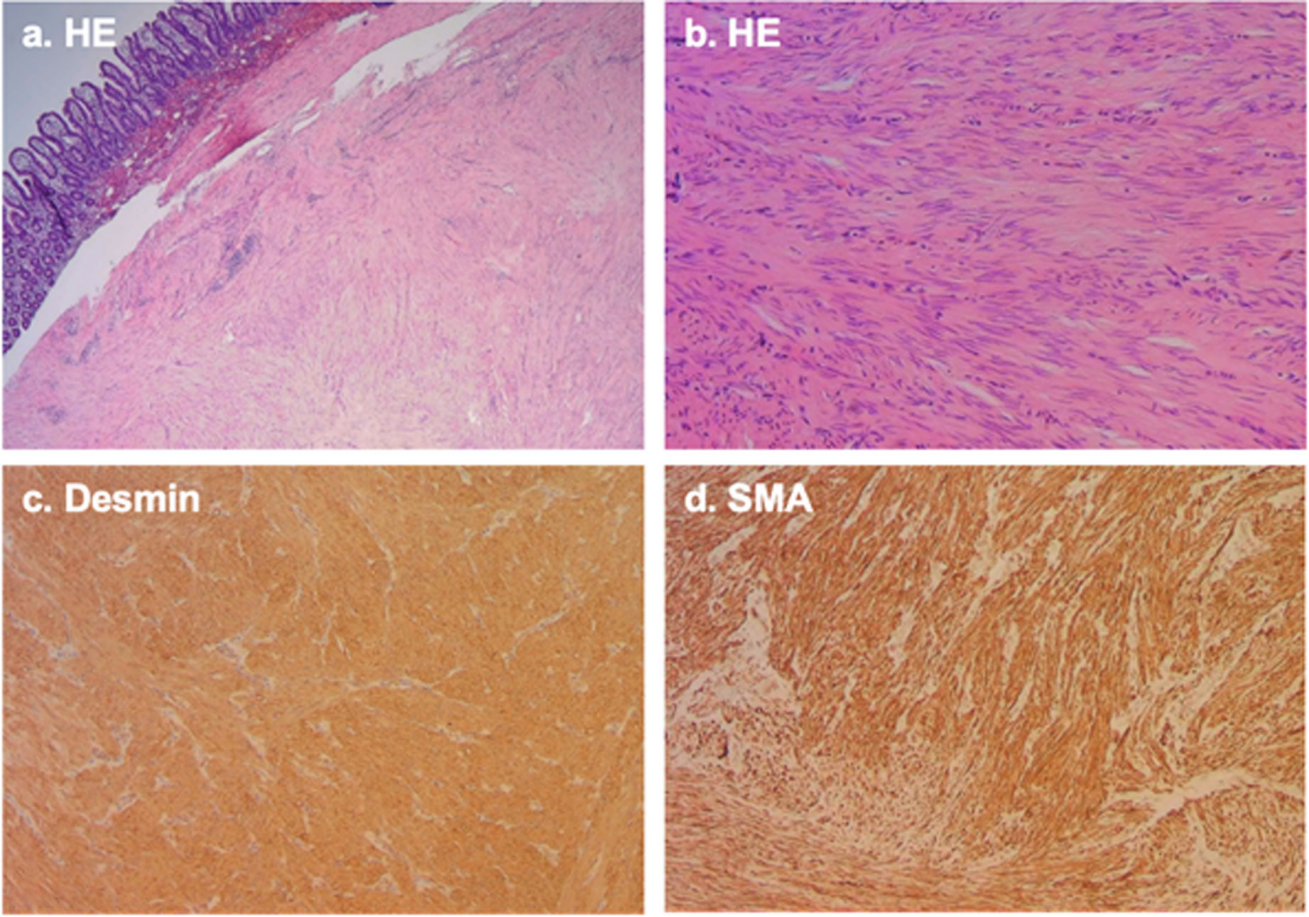
Immunohistochemical staining of a leiomyoma. (a and b) Hematoxylin and eosin (HE) staining in a leiomyoma. The nuclei of leiomyomas are elongated oval with rounded ends (cigar‐shaped). The chromatin is loose. A fishlike orientation of the cells is often seen. Mitoses are very sporadic, which allows differentiation from leiomyosarcoma. (c) Cytoplasmic positivity for desmin. (d) Cytoplasmic positivity for SMA (smooth muscle Actin) to show that the tumor contains smooth muscle cells. Magnifications: (a and c) 1×, (b and d) 10×.

Microscopically, leiomyomas are primarily characterized by bundles of smooth muscle cells (see Figure 1) interspersed with hyaline cartilage and no evidence of high mitosis [8]. Immunohistochemically, leiomyomas are negative for cluster of differentiation (CD)117 and CD34.

## 2 Case History

A 61‐year‐old man presented to our emergency department with anorectal bleeding, shortness of breath and general weakness. He reported a significant amount of bright red blood in his stool.

A year earlier, the patient had experienced a similar episode, during which he collapsed and was admitted to the emergency room of another hospital with severe iron‐deficiency anemia. During that previous admission, gastroscopy, and colonoscopy showed no acute source of bleeding. However, capsule endoscopy revealed a lesion in the small intestine without active bleeding. The etiology of the lesion was described as inconclusive but was interpretated as an angiodysplasia. Due to the location which was endoscopically hard to reach and the self‐limiting nature of the bleeding, no further treatment was pursued.

## 3 Methods (Investigations and Treatments)

During the current stay in our hospital, the patient reported no pain, and the physical examination was inconclusive, except for the rectal examination, where hematochezia presented on the glove.

Abdominal ultrasound showed fluid‐filled bowel loops without acute pathology. Complete ileocolonoscopy did not reveal an active source of bleeding, although the mucosa of the terminal ileum and colon was coated with a significant amount of bright red blood. As the ultrasound indicated no signs of obstruction and an angiodysplasia was suspected during a similar episode at another hospital a year ago, we decided to initiate a capsule endoscopy immediately after the colonoscopy to further investigate the source of bleeding.

**FIGURE 2:**
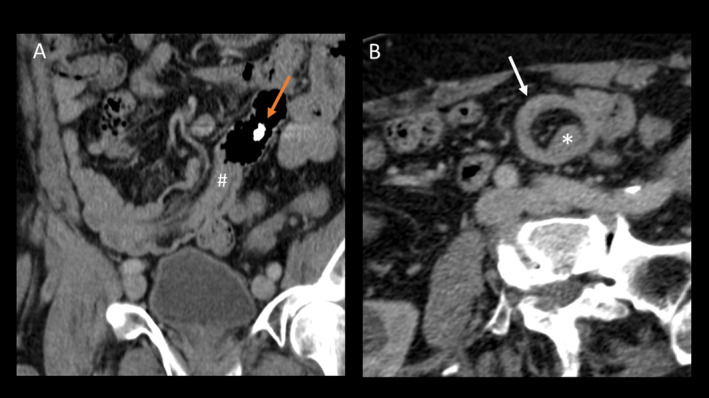
Contrast enhanced abdominal computed tomography (90 mL ACCUPAQUE^TM^ 300 mg) shows the intestinal intussusception (#) in the coronal plane together with the stuck video capsule (orange arrow in A). Axial images resemble the typical target sign appearance (white arrow) of the invaginated ileum (* in B).

Over the next few hours, the hemoglobin level dropped from 12 to 5.3 g/dL. An emergency CT scan revealed a long intussusception of the small bowel, with the capsule located just before the intussusception (see Figure 2). The patient received a transfusion of red blood cells concentrates and was transferred to the surgical team for an ileal segment resection.

The surgery began laparoscopically. A pneumoperitoneum was established at 14 mmHg. The optic was inserted supraumbilical, and 5 mm working trocars were inserted in the left lower quadrant and along the midline above the symphysis. The intussusception was found 1 m orally of the ileocoecal junction in the ileum. The cause was a 3 × 4 cm tumor in the small intestine with retraction of the antimesenteric wall of the small intestine. For further inspection and therapy the supraumbilical minilaparotomy was extended to approximately 10 cm. It became apparent that the intussusception had already resolved partially. Therefore, a Hutchinson maneuver was not necessary. The endoscopic capsule could not be palpated in the entire small intestine, so it was assumed that it had passed into the colon. A few hardened but not enlarged lymph nodes could be found in the lymphatic drainage area. Therefore, a malignant process was suspected, and the patient received an oncologic small bowel segmental resection with lymphadenectomy and end‐to‐end ileoileostomy. The total length of the resected ileum was approximately 40 cm.

## 4 Outcome and Results

The postoperative course was uneventful, and the patient recovered quickly, with no signs of recurrent bleeding. The capsule was found in the stool after surgery. A chest CT scan showed no evidence of distant metastasis. Histopathological analysis revealed a leiomyoma. Immunohistochemical examination was positive for desmin and SMA. CD117, S100, MCK, D0G1 were negative. CD34 showed numerous vessels within the spindle cell proliferate. Ki‐67 was positive in < 1% of the proliferating cells. The lymph nodes were unremarkable.

## 5 Discussion

Usually, leiomyomas remain asymptomatic. Occasionally, they may cause massive hemorrhage or occult blood, which can lead to chronic iron deficiency anemia. A less common manifestation is small bowel obstruction caused by intussusception, leading to mechanical ileus, and necrosis. Only a limited number of such cases have been reported in the literature so far [5, 9, 10, 11, 12, 13, 14, 15, 16, 17, 18, 19, 20, 21]. Instead, similar cases usually describe inflammatory fibroid polyps or other causes as the lead point [22, 23]. Intussusception caused by leiomyomas typically presents with acute onset of severe abdominal pain, nausea and vomiting, while hematochezia is described rarely [5].

The patient's lack of pain and absence of signs of stricture or ileus on the abdominal ultrasound made intussusception initially unlikely. However, the CT scan later confirmed the diagnosis, showing that the passage of the video capsule was hindered by the intussusception. However, when laparoscopic surgery was performed the intussusception had already partially resolved again and the capsule had passed through. The intermittent nature of the intussusception and the previous self‐limiting episode suggest that altered bowel mechanics caused by the leiomyoma led to recurrent, self‐resolving intussusceptions. We only found a few other cases which present chronic/intermittent intussusception due to leiomyomas [2, 16, 21], however these cases did not describe any bleeding.

As a key learning, a stenosis should always be excluded before a capsule is ingested to avoid occlusion and ileus by the capsule. In this case we initiated a capsule endoscopy right after the inconclusive colonoscopy as an angiodysplasia had been suggested as the cause of a similar episode at another hospital a year earlier. Retrospectively, it would have been preferable to first check for bowel obstruction using an abdominal CT scan. If an obstruction is present, a small bowel endoscopy can be used for tattooing the lesion to facilitate laparoscopic surgery.

In cases where imaging cannot be used to exclude an obstruction, most suppliers offer biodegradable dummy capsules to test whether the capsule can safely pass the gastrointestinal tract.

For the treatment decision, it is highly relevant to distinguish benign leiomyomas from malignant leiomyosarcomas [8]. While leiomyomas only need a simple resection (if symptomatic), leiomyosarcomas require an oncologic resection with the greatest possible margin of removal due to their aggressive growth. Leiomyosarcomas are usually soft and often hemorrhagic. Microscopically, they show abnormal mitotic figures, and coagulative tumor cell necrosis. Determination of the proliferation index Ki‐67 is essential to distinguish leiomyomas from leiomyosarcomas [24] and to specify tumor grading.

Imaging modalities useful for diagnosing small bowel pathologies include abdominal ultrasound, CT scan, or MRI. On CT images, intussusceptions typically look similar to a “target” mass when the beam is perpendicular to the longitudinal axis of the intussusception, and as a “sausage‐shaped” mass when the beam is parallel or oblique to the longitudinal axis [25].

## Author Contributions

**Leonard Fehring:** conceptualization, funding acquisition, investigation, methodology, project administration, supervision, visualization, writing – original draft, writing – review and editing. **Sarah Krüger:** writing – review and editing. **Sophia Vorreuther:** writing – review and editing. **Sarah Sass:** writing – review and editing. **Sophie Vieser:** writing – review and editing. **Hendrik Brinkmann:** writing – review and editing. **Lars Kamper:** visualization, writing – review and editing. **Maximilian Ackermann:** visualization, writing – review and editing. **Christian Prinz:** writing – review and editing.

## Consent

The manuscript and images do not contain any information that allow the identification of the patient. Still, we received written consent of the patient to publish the history and images presented in this case report.

## Conflicts of Interest

The authors declare no conflicts of interest.

## Data Availability

All the findings are present within the manuscript.

## References

[1] P. Marsicovetere , S. J. Ivatury , B. White , and S. D. Holubar , “Intestinal Intussusception: Etiology, Diagnosis, and Treatment,” Clinics in Colon and Rectal Surgery 30, no. 1 (2017): 30–39. 28144210 10.1055/s-0036-1593429 PMC5179276

[2] D. G. Begos , A. Sandor , and I. M. Modlin , “The Diagnosis and Management of Adult Intussusception,” American Journal of Surgery 173, no. 2 (1997): 88–94. 9074370 10.1016/S0002-9610(96)00419-9

[3] L. K. Eisen , J. D. Cunningham , and A. H. Aufses, Jr. , “Intussusception in Adults: Institutional Review,” Journal of the American College of Surgeons 188, no. 4 (1999): 390–395. 10195723 10.1016/s1072-7515(98)00331-7

[4] M. Tarchouli and A. Ait Ali , “Adult Intussusception: An Uncommon Condition and Challenging Management,” Visc Med 37, no. 2 (2021): 120–127. 33981752 10.1159/000507380 PMC8077547

[5] R. N. Nuruddin , A. H. Zakariya , and D. Abdullah , “Adult Jejunojejunal Intussusception. A Case Report,” Australasian Radiology 31, no. 3 (1987): 269–270. 3435344 10.1111/j.1440-1673.1987.tb01828.x

[6] M. Miettinen , J. Kopczynski , H. R. Makhlouf , et al., “Gastrointestinal Stromal Tumors, Intramural Leiomyomas, and Leiomyosarcomas in the Duodenum: A Clinicopathologic, Immunohistochemical, and Molecular Genetic Study of 167 Cases,” American Journal of Surgical Pathology 27, no. 5 (2003): 625–641. 12717247 10.1097/00000478-200305000-00006

[7] D. K. Blanchard , J. M. Budde , G. F. Hatch, III , et al., “Tumors of the Small Intestine,” World Journal of Surgery 24, no. 4 (2000): 421–429. 10706914 10.1007/s002689910067

[8] S. S. Gill , D. M. Heuman , and A. A. Mihas , “Small Intestinal Neoplasms,” Journal of Clinical Gastroenterology 33, no. 4 (2001): 267–282. 11588539 10.1097/00004836-200110000-00004

[9] S. G. Romanini and J. C. Ardengh , “Adult Jejunojejunal Intussusception Caused by Small Bowel Leiomyoma,” Cureus 16, no. 7 (2024): e63587. 39087153 10.7759/cureus.63587 PMC11290382

[10] V. J. Mansberg , G. Mansberg , and B. D. Doust , “Jejunojejunal Intussusception Secondary to Leiomyoma,” Australasian Radiology 40, no. 1 (1996): 72–74. 8838894 10.1111/j.1440-1673.1996.tb00350.x

[11] L. Iliescu , L. Toma , M. Grasu , and V. Herlea , “A Rare Case of Intestinal Obstruction,” Journal of Gastrointestinal and Liver Diseases 22, no. 3 (2013): 250. 24078977

[12] O. Sunamak , I. Karabicak , I. Aydemir , et al., “An Intraluminal Leiomyoma of the Small Intestine Causing Invagination and Obstruction: A Case Report,” Mount Sinai Journal of Medicine 73, no. 8 (2006): 1079–1081. 17285198

[13] M. Guercio , E. Hanisch , and V. Paolucci , “Invagination of the Small Intestine Due to Leiomyoma. A Case Report of Echographic Diagnosis,” Minerva Chirurgica 49, no. 1–2 (1994): 95–97. 8208477

[14] M. Draskovic , S. Misovic , G. Kronja , J. Krsic , A. Tomic , and M. Sarac , “Jejuno‐Jejunal Intussusception in Adults Secondary to Submucosal Leiomyoma,” Medicinski Pregled 58, no. 7–8 (2005): 405–409. 16296586 10.2298/mpns0508405d

[15] Y. Ueda , M. Tominaga , N. Nishijima , et al., “Laparoscopy for Adult Intussusception Caused by Leiomyoma of the Jejunum,” Journal of Clinical Gastroenterology 27, no. 3 (1998): 255–256. 9802456 10.1097/00004836-199810000-00015

[16] A. Alvarez Sanchez , C. Ciriza de los Ríos , J. García Cabezas , et al., “Leiomyoma‐Dependent Ileal Invagination as a Cause of Intermittent Intestinal Obstruction,” Anales de Medicina Interna 12, no. 10 (1995): 505–507. 8519944

[17] S. C. Schmidt , J. M. Langrehr , and E. Rivas , “Invagination of the Ileum as the Etiology of Acute Abdomen in Adults,” Zentralblatt für Chirurgie 125, no. 11 (2000): 907–909. 11143515 10.1055/s-2000-10058

[18] W. Bruins Slot , S. A. Koopal , and J. F. Keuning , “Intussusception in Disseminated Peritoneal Leiomyomatosis,” Nederlands Tijdschrift voor Geneeskunde 136, no. 32 (1992): 1564–1566. 1528284

[19] R. Fischbach , W. Gross‐Fengels , and U. Hesse , “Chronic Small Bowel Invagination Due to Leiomyoma,” Röntgen‐Blätter 43, no. 6 (1990): 248–250. 2371513

[20] R. Weiner , R. Haupt , and C. Klemm , “Ileus Caused by Ileocecal Invagination of a Leiomyofibroma,” Zentralblatt für Chirurgie 113, no. 23 (1988): 1540–1543. 3239292

[21] M. Hladik and J. Klestil , “Leiomyoma With Chronic Invagination of the Small Intestine,” Ceskoslovenská Radiologie 20, no. 4 (1966): 249–252. 5947620

[22] H. T. Chiu , H. Yen , Y. S. Weng , et al., “Combined Ileoileal and Ileocolic Intussusception Secondary to Inflammatory Fibroid Polyp in an Adult: A Case Report,” Medicina (Kaunas, Lithuania) 58, no. 2 (2022): 310. 10.3390/medicina58020310 PMC8874661 35208633

[23] O. Michael , K. Derick , and O. Ponsiano , “Gastroduodenal Intussusception as a Rare Cause of Pancreatitis in a Young Female Ugandan: A Case Report,” International Journal of Surgery Case Reports 89 (2021): 106632. 34844196 10.1016/j.ijscr.2021.106632 PMC8636807

[24] M. Sanci , C. Dikis , S. Inan , E. Turkoz , N. Dicle , and C. Ispahi , “Immunolocalization of VEGF, VEGF Receptors, EGF‐R and Ki‐67 in Leiomyoma, Cellular Leiomyoma and Leiomyosarcoma,” Acta Histochemica 113, no. 3 (2011): 317–325. 20106509 10.1016/j.acthis.2010.01.001

[25] V. Valentini , G. L. Buquicchio , M. Galluzzo , et al., “Intussusception in Adults: The Role of MDCT in the Identification of the Site and Cause of Obstruction,” Gastroenterology Research and Practice 2016 (2016): 5623718. 26819606 10.1155/2016/5623718 PMC4706914

